# Observation and an Explanation of Breakdown of the Quantum Hall Effect

**DOI:** 10.6028/jres.095.009

**Published:** 1990

**Authors:** M. E. Cage, D. Y. Yu, G. Marullo Reedtz

**Affiliations:** National Institute of Standards and Technology, Gaithersburg, MD 20899

**Keywords:** acoustic phonon, breakdown of the dissipationless state, inter-Landau level scattering, population inversion, quantum Hall effect, two-dimensional electron gas

## Abstract

We observe a spatially localized breakdown of the nearly dissipationless quantum Hall effect into a set of discrete dissipative states in wide, high-quality GaAs/AlGaAs samples. The phenomenon can be explained by an extension of the quasi-elastic inter-Landau level scattering model of Eaves and Sheard.

## 1. Introduction

The integral quantum Hall resistance [1] *R*_H_(*i*)=*V*_H_(*i*)/*I_x_*=*h*/(*e*^2^*i*) is observed when the longitudinal resistance *R_x_* = *V_x_*/*I_x_* of the two-dimensional electron gas is very small. Here *V*_H_(*i*) is the Hall voltage of the *i*th plateau, *i* is an integer, and *I_x_* is the current through the sample. There is a critical current *I*_c_ above which *R_x_* rapidly increases by several orders of magnitude [2,3]. *R_x_* becomes finite as one approaches the critical current. This is referred to as breakdown.

We present results of a breakdown experiment in which sets of discrete *V_x_* signals are observed. We propose an explanation based on an extension of the quasi-elastic inter-Landau level scattering (QUILLS) mechanism of Eaves and Sheard [4].

## 2. The Experiment

**Figure 1:**
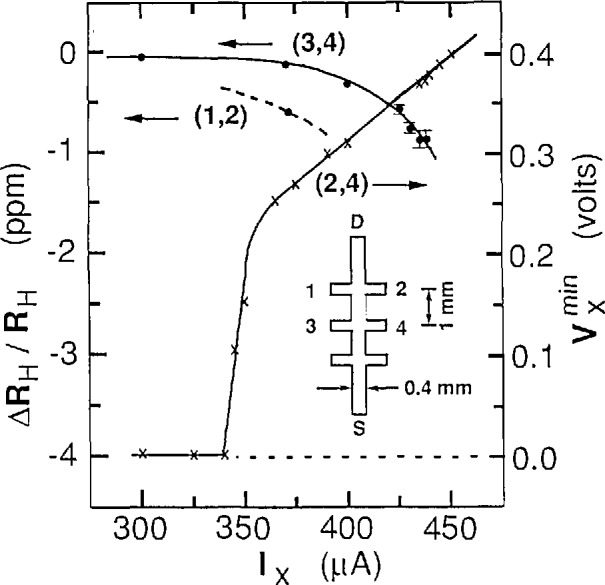
Current dependence of *∆R*_H_/*R*_H_ and $V_{x}^{\min}$ for the *i* = 4 plateau of the GaAs(7) sample at 1.2 K. The inset shows the sample geometry.

Our samples [3] were GaAs/Al*_x_*Ga_1−_*_x_*As heterostructures grown by molecular beam epitaxy with *x* = 0.29. The samples are designated as GaAs(7) and GaAs(8). They have zero magnetic field mobilities of ~100,000 cm^2^/(V·s) at 4.2 K. They exhibit excellent integral quantum Hall effect properties. The inset of figure 1 shows the geometry of the samples. They are 4.6 mm long and 0.4 mm wide. Contact was made to the two-dimensional electron gas at points 1, 2, 3, and 4, as well as the source S and the drain D.

An important feature of the GaAs(7) sample is that the breakdown occurs somewhere within the longitudinal shaded region between the middle Hall probe pair 3,4 and the outer Hall probe pair 1,2 of figure 1, but not within the transverse region of either Hall probe pair. This crucial fact can be explained by examining figure 1. The minimum *V_x_* signal, $V_{x}^{\min}$ measured between probe pairs 2 and 4, increases by a factor of 10^7^ between *I_x_* =25 *μ*A and *I_x_* = 370 *μ*A, but the quantized Hall resistance *R*_H_ for the *i*=4 plateau decreases by only one part in 10^7^ for probe pair 3,4 at 370 *μ*A, and by only six parts in 10^7^ for probe pair 1,2. These changes in *R*_H_ are only about 0.01% of what was expected from the mixing of *V_x_* into *V*_H_ due to the known misalignment of the Hall probes. We therefore know the general region where the breakdown occurs, and that the *i* = 4 Hall resistance is accurately quantized on both sides of the spacial breakdown region.

**Figure 2:**
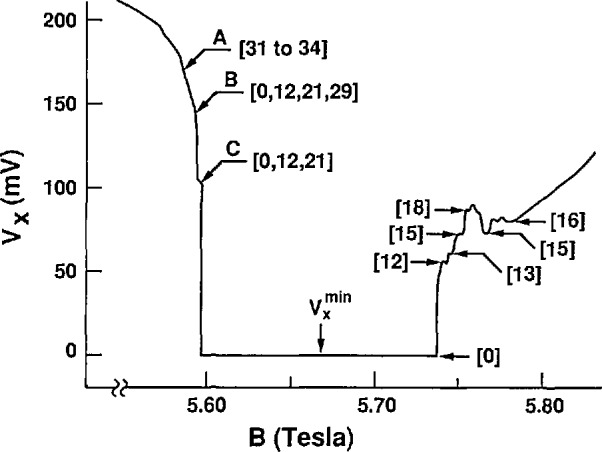
Time-averaged *V_x_* signals for the *i* = 4 plateau of GaAs(7) at 1.2 K and 300 *μ*A. The number of inter-Landau level transitions (*N′−N*) is indicated in brackets.

**Figure 3:**
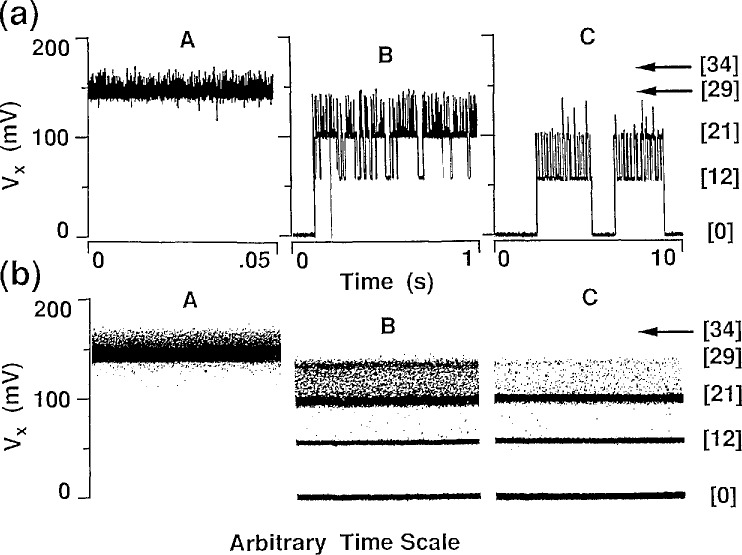
(a) Digital oscilloscope recordings of the time-dependent *V_x_* signals at the positions labeled A, B, and C in figure 2. (b) Digital oscilloscope recordings of the time-averaged distribution of the values of *V_x_* at the positions A, B, and C.

The critical current at which $V_{x}^{\min}$ starts to rise abruptly is *I*_c_ = 340 *μ*A. Figure 2 shows a *V_x_* vs *B* curve for GaAs(7) at *I_x_* = 300 *μ*A, well below that critical current. This curve exhibits reproducible structures. Figure 3, (a) and (b), respectively, show digital oscilloscope displays of the time-dependence and time-averaged distributions of the values of *V_x_* obtained at points A, B, and C of figure 2. There are clearly distinct dc voltage levels and switching between levels. Figure 3 clearly shows that the character of these levels changes with magnetic field. Also, *V_x_* is in only one state at any given time. It remains in that state until electrical noise or other noise processes induces it to switch to another state. Similar switching between dc voltage levels occurs in the GaAs(8) sample. The *V_x_* curve of figure 2 masks this switching by displaying time-averaged values of these dc voltage levels.

We make an initial attempt to interpret our observations by using a modified version of the QUILLS model of Eaves and Sheard [4] because that is a model which seems to provide a satisfactory explanation. Eaves and Sheard used their model to interpret the experimental data of Bliek et al. [5] who made breakdown measurements on GaAs/AlGaAs samples in which Hall potential probe sets were placed on either side of a 1 *μ*m wide constriction. Their *V_x_* vs *B* curves show features similar to our data, and their Hall voltages were also quantized. Our samples have no geometrical constriction; they are instead 400 *μ*m wide. But we propose that the nearly dissipationless conduction channel is very narrow in our samples as the critical current is approached, and that the conduction channel becomes entirely pinched-off and dissipative above the critical current. Our samples therefore may have effective geometries similar to those of Bliek et al. [5] near breakdown. We assume the global current-carrying equipotentials of the percolation model [6], as strongly suggested by experiments which measured the potential distribution across quantum Hall devices [7].

## 3. The QUILLS Model

**Figure 4:**
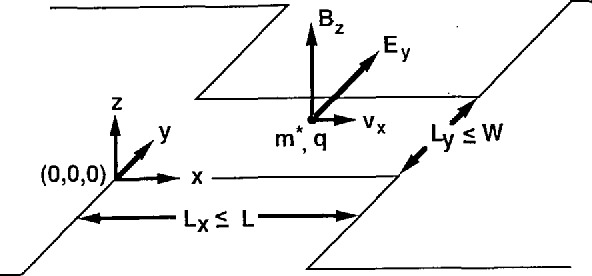
Motion of a charged particle through a constriction. The dynamical length and width *L_x_* and *L_y_* may be smaller than the physical length and width *L* and *W*.

To explain our data we first extend the QUILLS model of Eaves and Sheard [4] to include transitions between non-contiguous Landau levels. Particles of positive charge *q* and reduced mass m* (0.068 times the free electron mass in GaAs) move with velocity *υ_x_* in the positive *x* direction through a constriction of average length *L_x_* and average width *L_y_* as shown in figure 4. The quantities *L_x_* and *L_y_* can be less than the length *L* and width *W of* a geometrical constriction. The average electric field within the constriction and the average magnetic field is *E_y_* and *B_z_*, respectively. Therefore, *υ_x_*=*E_y_/B_z_*. The Hamiltonian for this system, neglecting spin-splitting and scattering, is $\hat{H}=\left(1/2m*\right){\left(\hat{p}-qA\right)}^{2}+qV_{y}$ in SI units, where $\hat{p}$ is the momentum operator *−iℏ∇, A* is the magnetic vector potential and *V_y_* = − *yE_y_*. Using the Landau gauge *A_x_* = − *yB_z_* and *A_y_*=*A_z_*=0, one obtains the normalized eigenfuctions ψ and energy eigenvalues C to Schodinger’s equation $\hat{H}\psi =C\psi$:

ψN,kx(x,ξ)=1(Lx) 1/2eikxx1(2NN!) 1/21(π) 1/4e−ξ22HN(ξ)ψN,kx(x,ξ)=1(Lx) 1/2eikxxΦN(ξ)CN(y0)=(N+12)ℏωc−qy0Ey+12m*υx2,

where *N* is the Landau level number, *ω*_c_=qB_z_/m* is the cyclotron angular frequency, *ℓ_B_*=(ℏ/*qB_z_*)^1/2^ is the magnetic length, $y_{0}=\left(\upsilon_{x}/\omega_{c}-\ell_{B}^{2}k_{x}\right)$ is the *y* coordinate of the center of motion of each cyclotron orbital, *k_x_* = *2πN_k_/L_x_* is the *x* component of the wavevector of a cyclotron orbital state (*N,Nk*), *N_k_* is the integer quantum number of the wavevector of that state, ξ = (*y − y*_0_)/*ℓ_B_*, and *H_N_*(ξ) is a Hermite polynomial.

**Figure 5:**
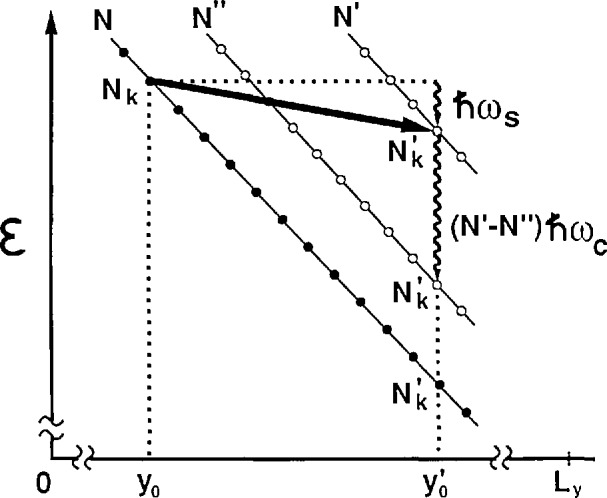
Total energy eigenvalues $C_{N}$ as a function of position *y* across the constriction for each Landau level *N*. Every eigenvalue of level *N* has a unique quantum number *N_k_*. The figure shows a QUILLS transition from level *N* to *N′* and an associated acoustic phonon. The decay, either directly down to level *N* or through an intermediate level *N*″, and its associated optical phonon, are also shown.

Figure 5 shows the energy eigenvalues for Landau level *N* as a function of *y*. The slope of the lines is *−qEy*. The eigenvalues are equally spaced, with separation $\Delta y_{0}=2\pi \ell_{B}^{2}/L_{x}$. The maximum number of allowed extended states (*N,N_k_*) is (*qB_z_/h)L_x_L_y_* for level *N*. The total number of allowed extended states per unit area is *n_s_=i*(*qB_z_/h*).

If *L_y_* becomes small enough and *E_y_* large enough then it may be possible for a particle to make a transition from state (*N,N_k_*) to an empty state $\left(N^{\prime },N_{k}^{\prime }\right)$ at a lower total energy, as shown in figure 5. The particle moves across the sample in this model from position *y*_0_ to $y_{0}^{\prime }$. Energy and momentum must be conserved in the transition. Therefore, an acoustic phonon of wavevector

$$K_{x}=\frac{2\pi }{L_{x}}\left(N_{k}-{N^{\prime }}_{k}\right)=\frac{\omega }{\left(\upsilon_{x}-\upsilon_{s}\right)}\left(N^{\prime }-N\right)$$ (1)

and energy $C_{\text{phonon}}=\hbar \omega_{s}=\hbar \upsilon_{s}K_{x}$ is emitted in the *x* direction, where *υ*_s_ is the velocity of sound in that medium (~2.47 × 10^3^ m/s in GaAs[8]).

**Figure 6:**
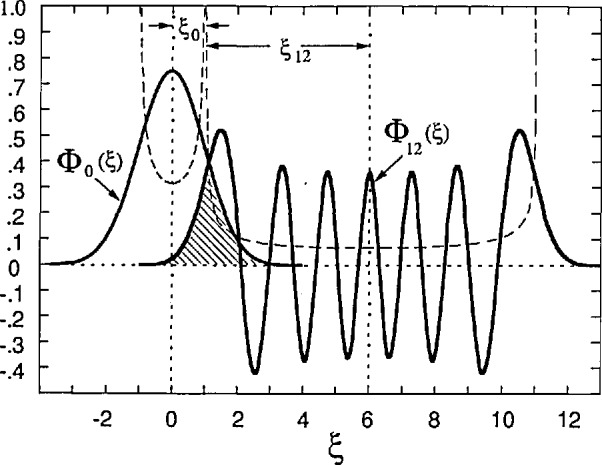
The wavefunctions Φ_0_(ξ) and Φ_12_(ξ), shown overlapping at the classical harmonic oscillator separations ξ_0_ and ξ_12_. The dashed lines represent the classical harmonic oscillator probability distributions.

Only transitions in which the ratio $\left(y_{0}^{\prime }-y_{0}\right)L_{x}/\left(2\pi \;\ell_{B}^{2}\right)$ is an integer number $\left(N_{k}-N_{k}^{\prime }\right)$ are allowed. One can obtain an equation for $\left(N_{k}-N_{k}^{\prime }\right)$ by estimating the value of $\left(y_{0}^{\prime }-y_{0}\right)$. This is accomplished by noting that the spatial extent of $\psi_{N,k_{x}}\left(x,\xi \right)$ or Φ*_N_*(ξ) is approximated well by the amplitude of motion of a classical harmonic oscillator: $A_{N}=\ell_{B}\sqrt{2N+1}$. Transitions can commence when the wavefunctions just begin to significantly overlap:

$$\left({y^{\prime }}_{0}-y_{0}\right)\approx \left(A_{N}+{A^{\prime }}_{N}\right)=\ell_{B}\xi_{c\ell }^{N,N^{\prime }}$$ (2)

where $\xi_{c\ell }^{N,N^{\prime }}=\sqrt{2N+1}+\sqrt{2N^{\prime }+1}$ Figure 6 shows such an overlap between Φ_0_(ξ) and Φ_12_(ξ). Thus

$$\left(N_{k}-{N^{\prime }}_{k}\right)\approx \frac{L_{x}}{2\pi \ell_{B}}\xi_{c\ell }^{N,N^{\prime }}.$$ (3)

There is another condition for the transition: (*N′−N*) must also be an integer. From conservation of energy, the electric field *E_y_* = *V*_H_/*L_y_* is

$$E_{y}=\left[\left(N^{\prime }-N\right)\hbar \omega_{c}+\hbar \omega_{s}\right]/q\left({y^{\prime }}_{0}-y_{0}\right).$$ (4)

The simultaneous integer conditions $\left(N_{k}-N_{k}^{\prime }\right)$ and (*N′−N*), given by eqs (3) and (4), occur only at particular values of the current and magnetic field. There may be many intervening Landau levels between *N* and *N′* in which these conditions are not satisfied. Notice that the filled states of Landau level *N* are at a higher total energy than those of the unoccupied *N′* level. The large Hall electric field *E_y_* has induced a population inversion for QUILLS transitions.

After the particles make the *N* to *N′* QUILLS transitions they then decay, by optical phonon emission [9], back down to the original Landau level *N*, either directly or by cascading through intermediate levels *N*″, as shown in figure 5. The optical phonon transitions probably occur just outside of the constriction. One can calculate the voltage signal *V_x_* from the electrical power dissipated, $P=I_{x}^{2}R_{x}=I_{x}V_{x}$, when the particles return to the lowest Landau level during the average time taken to traverse the constriction:

$$V_{x}=\frac{1}{q}\frac{n^{\prime }}{n_{s}}\left[\left(N^{\prime }-N\right)\hbar \omega_{c}+\hbar \omega_{s}\right],$$ (5)

where *I_x_=qn_s_υ_x_L_y_*, and *n′/n_s_* is the fraction of conducting particles that make the transition. We assume both spin states can undergo transitions. Therefore *N* is 0 and *n*′/*n*_s_≤1 for the *i=2* plateau. *N* is either 0 or 1 and *n*′/*n*_s_≤1/2 for the *i*=4 plateau. If all the electrons make the transition, then *n*′*/n*_s_ is 1 and 1/2 for the *i*=2 and 4 plateaus.

## 4. Analysis of Our Data

We next apply this QUILLS model to our *V_x_* vs *B* data at 300 *μ*A. Equation (5) is used first to obtain the values of (*N′−N*) by ignoring the small term *ℏω*_s_. The electric field *E_y_* is then calculated from eqs (4) and (2) for specific values of *V_x_* and *B_z_. E_y_* ranges between 1.6−3.9×10^6^ V/m across the quantized Hall resistance plateau. The constriction width *L_y_* = *V*_H_*/E_y_* = *I_x_R_H_/E_y_* varies between 0.5−1.2 *μ*m across the plateau, and is narrower on either side of the *V_x_≈O* region. The range of *υ_x_=E_y_/B_z_* is 2.8−7.0×10^5^ m/s across the plateau, and *υ_x_* is ~200*υ*_s_. Also, *ℏω_t_*=*ℏυ*_s_*K_x_*, calculated from eq (1), is ~0.5% of (*N′−N*)*ℏω_c_*. The dissipation of the QUILLS transitions is therefore very small indeed. The current density *J_x_=I_x_/L_y_=E_y_*/(*h*/4*q^2^*) varies between 240–600 A/m. This is an astonishingly large number compared with that found in [5], but the current has a better opportunity of finding a dissipationless path in wide high-quality samples. Also, *qV*_H_*≈*200*ℏω*_c_. This is very large compared with our deduced (*N′−N*)*ℏω*_c_ values, and is in an entirely different regime than that of Kirtley et al. [10] who found small integer values of *qV*_H_*/ℏω*_c_. $\left(N_{k}-N_{k}^{\prime }\right)$ is the only quantity that can not be deduced from the data because the length of the constriction *L_x_* is unknown. If *L_x_* is assumed to be comparable to the width, i.e., about 1 *μ*m, then $\left(N_{k}-N_{k}^{\prime }\right)\approx 90$ from eq (3). Finally, eq (5) predicts that the quantity (*n*_s_/*n′*)(m*/*ℏ*)*V_x_*/[(*N′−N*)*B_z_*] should be nearly equal to 1. This quantity is within 3% of 1 for the (*N′−N*) transitions shown in figure 2, and is within our experimental accuracy. All of the electrons in Landau level *N* apparently make the transition to level *N*′.

Multiple values of *V_x_* sometimes occur at certain values of *B_z_*, as for example at points B and C of figures 2 and 3. In such cases the above equations yield a different value of *L_y_* for each value of *V_x_*. A more realistic approach in those situations is to use the smallest value of *L_y_* obtained from the largest value of *V_x_* for that magnetic field. The values of *L_y_* and *E_y_* are then constant for all QUILLS transitions observed at a given *B_z_* and *I_x_*. With these assumptions, the values of ξ,*^N,N^′* for the smaller *V_x_* transitions are then less than the classical values $\sqrt{2N+1}+\sqrt{2N^{\prime }+1}$. This presents no problem because the overlap integral between Φ*_N_*(ξ) and Φ*_N_*_′_(ξ′) becomes somewhat larger inside of the classical value and then falls off slowly over a wide region as ξ,*^N,N^′* is reduced.

The features labeled with the (*N′−N*) values 12, 13, 15, 16, and 18 on the right hand side of the *V_x_* curve of figure 2 have very stable single-valued signals. This is not always the case; for example, we see switching about the (*N′−N*)=12 transition at position C and about the (*N′−N*)=21 transition at position B. We believe that the switching is noise-induced.

We clearly observe discrete levels of *V_x_* in figure 3. It is difficult to understand why the overlap of the rather broad wavefunctions and the integer requirements of the apparently large values of (*N′−N*) and $\left(N_{k}-N_{k}^{\prime }\right)$ would by themselves lead to preferential *N* to *N′* inter-Landau level transitions. An additional, unaccounted selection mechanism may be present.

One has to be careful about the definition of critical current. For example, in our experiment *V_x_* is large and there appears to always be dissipation at points B and C in figure 2. However, when looking at the discrete voltage states of those points in figure 3 we see that for one of those states (*V_x_*≈0) the dissipation is negligible. Therefore the critical current has not yet been exceeded.

## 5. Discussion

Our interpretation of the QUILLS model for breakdown is consistent with the experiment of Bliek et al. [5], who made physical constrictions of order 1 *μ*m width and 10*μ*m length. They observed step-like structures in their *V_x_* vs *B* curves and empirically found equations for the quantized values of *R_x_* at those steps and for the magnetic field values at which the structures occur. The empirical number *n_L_=L_x_*(*2qB_z_/h*)^1/2^ of Bliek et al. [5] corresponds to $n_{L}=\left(N_{k}-N_{k}^{\prime }\right){\left(4\pi \right)}^{1/2}/\xi_{c\ell }^{N,N^{\prime }}$ in our formulation, whereas Eaves and Sheard [4] predicted that $n_{L}=\left(N_{k}-N_{k}^{\prime }\right)$. The equation *R_x_=n_R_*(*n_L_/n_0_*)(*h/2e^2^*) of [5] corresponds to our interpretation of QUILLS if $n_{R}=\left(n^{\prime }/n_{s}\right)\xi_{c\ell }^{N,N^{\prime }}/{\left(\pi \right)}^{1/2}$ and *n*_0_=*n_s_L_x_L_y_*. Eaves and Sheard [4] predict that *n_R_* is an integer number. Neither *n_L_* nor *n_R_* are integers in our formulation.

In our interpretation of the data of Bliek et al. [5] the values of (*N′−N*) vary between 3 and 13 for their *i* = 2 plateau assuming, as they did, that their *V_x_* signal is not a time average of several Landau level transitions. They obtained values of *n_R_* between 2 and 19. We find that the effective constriction of their sample *L_y_* varies between about 0.2−0.5 *μ*m, significantly less than the 1 *μ*m physical width. If *L_x_* equals the physical 10.3 *μ*m length *L*, then $\left(N_{k}-N_{k}^{\prime }\right)$ ranges between about 650–990. *L_x_* is probably much less than *L*, yielding smaller values of $\left(N_{k}-N_{k}^{\prime }\right)$. Bliek et al. [5] found that *n_L_* varied between 528 and 607.

Sachrajda et al. [11] also used samples with a narrow constriction. They observed structures having the same triangular behavior as that of the critical current versus magnetic field plot of Bliek et al. [12]. They believed that this behavior did not agree with the QUILLS model. But we find from our data that the conduction channel narrows on either side of *V_x_*≈0, and because less current is required in a narrower channel to obtain the same electric field, our results show that the QUILLS model is consistent with that behavior.

It has been proposed [13,14] that breakdown is due to emission of phonons in a manner analogous to the Cherenkov effect if *υ_x_>υ*_s_. In the QUILLS interpretation of our data *υ_x_*≈200*υ*_s_ and the Cherenkov angle *θ*_c_=cos^−1^(*υ*_s_*/υ*_c_) varies between 89.5° and 89.8°. The dissipative voltage *V_x_* can be very small even when the velocity *υ_x_* is apparently quite large. The experiment of Bliek et al. [5] provides excellent proof that this may indeed be the case. Figure 1 of their paper shows that *V_x_* has structures on the sides of the *i* = 2 plateau and goes to zero in the center of the plateau for *I_x_*=45 *μ*A, *L_y_*≈1 *μ*m, and *B_z_*≈6.4 T. Therefore, *υ_x_*=*E_y_/B_z_*=(*I_x_R_H_*)/(*L_y_B_z_*)≈36.7υ_s_, and yet *V_x_* still goes to zero.

We have considered the acoustic phonon emission to be collimated in the *x* direction. However, the electron gas has a probability distribution in the *z* direction that extends over an interval *∆z* which is about 50 Å in GaAs/AlGaAs. Therefore the acoustic phonons may have *K_z_*~1/∆*z* components which are about 35% of the magnitude of the *K_x_* component in our experiment for the (*N′−N*)=12 transition. Phonon emission in the *x−z* plane would not have a significant effect on the results of this paper, but it might preclude the possibility of spontaneously emitted phonons stimulating the other phonons to make population-inverted QUILLS transitions in a manner analogous to photon emission of lasers.

## 6. Conclusions

We have observed spatially localized breakdown of the nearly dissipationless quantum Hall effect into a discrete set of dissipative states, and have interpreted these observations in terms of the QUILLS model. Theoretical analyses of QUILLS transition rates, similar to [13], and optical phonon decay rates would be very useful in determining the validity of QUILLS. Direct observation of the acoustic phonons would confirm the QUILLS process. There are other interesting features that could be studied: the decay process; the quantum heater property of *R_x_;* and the fact that two crossed resistances, *R*_H_ and *R_x_*, are both quantized. We suggest that QUILLS transitions may be the dominant breakdown mechanism in high-quality integral quantum Hall effect samples, and that the discrete *V_x_* signals are indicators of those transitions.
